# Neurobehavioral outcomes and associated risk factors in pediatric brain tumor survivors

**DOI:** 10.1007/s11060-026-05558-8

**Published:** 2026-04-11

**Authors:** Yuliang Wang, Wing Lam Chan, Fei Li, Jiaoyang Cai, Yin Ting Cheung, Eric Tsz Him Lai, Oscar Lok Kan Leung, Shiting Liang, Dennis Tak Loi Ku, Matthew Ming Kong Shing, Eric Chun Ho Fu, Jeffrey Ping Wa Yau, Anselm Chi Wai Lee, Evelyn Ruoyun Lu, Alex Wing Kwan Leung, Frankie Wai Tsoi Cheng, Wilson Wai Shing Ho, Zeng Gao, Ye Song, Stephenie Ka Yee Liu, Lucia Tsang, Ada Nga Yee Yuen, Tatia Mei Chun Lee, Godfrey Chi Fung Chan, Anthony Pak Yin Liu, Winnie Wan Yee Tso

**Affiliations:** 1 Department of Paediatrics & Adolescent Medicine, School of Clinical Medicine, LKS Faculty of Medicine, The University of Hong Kong, Pokfulam, Hong Kong China; 2 Department of Developmental and Behavioural Paediatric and Child Primary Care & Ministry of Education - Shanghai Key Laboratory of Children’s Environmental Health, Xinhua Hospital Affiliated to Shanghai Jiao Tong University School of Medicine, Shanghai, China; 3 Department of Hematology & Oncology, Shanghai Children’s Medical Center, Shanghai Jiao Tong University School of Medicine, Shanghai, China; 4 School of Pharmacy, Faculty of Medicine, The Chinese University of Hong Kong, Hong Kong, China; 5 Hong Kong Hub of Paediatric Excellence, The Chinese University of Hong Kong, Hong Kong, China; 6 Department of Psychiatry, School of Clinical Medicine, LKS Faculty of Medicine, The University of Hong Kong, Hong Kong, China; 7 Hong Kong Children’s Hospital, Kowloon, Hong Kong China; 8 Xuanwu Hospital, Capital Medicine University, Beijing, China; 9 Guangzhou Women and Children’s Medical Center, Guangzhou, China; 10 Child Assessment Service, Department of Health, Government of the HKSAR, Hong Kong, China; 11 Laboratory of Neuropsychology and Human Neuroscience, Department of Psychology, University of Hong Kong, Hong Kong, China; 12 InnoCentre of Clinical Neuropsychology, The University of Hong Kong, Hong Kong, China; 13 Division of Haematology/Oncology, The Hospital for Sick Children, Toronto, ON Canada; 14 The Duchess of Kent Children’s Hospital at Sandy Bay, Hong Kong, China

**Keywords:** Pediatric brain tumor survivors, Neurobehavioral outcomes, Emotional/behavioral difficulties, Cancer survivorship, Quality of life

## Abstract

**Purpose:**

Survival rates for children with brain tumors improve, highlighting the importance of understanding the long-term neurobehavioral outcomes because of its impact on children’s well-being and quality of life. This study investigated the prevalence of attention-deficit/hyperactivity disorder (ADHD), autism spectrum disorder (ASD) and emotional/behavioral difficulties in pediatric brain tumor survivors (PBTS), and identified the risks and protective factors on mental well-being.

**Methods:**

A territory-wide retrospective cohort included 274 PBTS registered in the Hong Kong Paediatric Haematology and Oncology Study Group Registry. In addition, a cross-sectional follow-up survey on mental well-being was completed by 107 PBTS during survivorship follow-up. Emotional/behavioral difficulties, health-related quality of life, parental stress, and sleep variables were assessed by the survey and benchmarked against previously published Hong Kong-based reference/community cohorts.

**Results:**

Among 274 PBTS, 10.6% had ADHD and 6.9% had ASD, which are significantly higher than the general pediatric population prevalence. They had more emotional/behavioral symptoms, higher parental stress, and poorer quality of life. Younger age at diagnosis, seizure history, and supratentorial tumors were linked to more difficulties. Radiotherapy was associated with reduced quality of life. Better sleep correlated with fewer ADHD and emotional symptoms.

**Conclusion:**

PBTS had increased risk of ADHD and ASD, and are more vulnerable to peer-relationship difficulties, poorer mental health, and quality of life. Improving sleep could be key to reducing neurobehavioral challenges. Implementing routine neurobehavioral monitoring, including sleep assessments, is crucial for enhancing survivorship care and overall well-being.

**Supplementary Information:**

The online version contains supplementary material available at 10.1007/s11060-026-05558-8.

## Introduction

Pediatric brain tumors are the most common type of solid tumors, and only second to leukemia as the main cause of pediatric malignancies [1]. In the most recent report by the Central Brain Tumor Registry of the United States [2], the estimated global incidence of primary malignant brain and other central nervous system (CNS) tumors was approximately 3.5 cases per 100,000 population in 2020 [2]. With advancements in cancer treatments, the 5-year survival rate of pediatric brain tumor survivors (PBTS) has improved significantly [3], reaching 35.7% for those with malignant CNS tumors and 91.8% for those with a non-malignant brain tumors [4]. Despite better survival rates, PBTS are prone to neurocognitive sequelae that can profoundly affect their quality of life [5–7]. Emerging evidence shows that PBTS treated for brain tumors experience executive function impairment, psychosocial adjustment difficulties [8] and behavioral challenges [9]. A meta-analysis conducted by our team showed that PBTS are also at risk of inattention, and psychosocial and emotional problems [10].

Existing studies on the sequalae of PBTS often focus on the neurocognitive outcomes(e.g., attention, memory, processing speed, and executive function) [6, 11, 12], with much less attention paid to effects of the tumor and treatments on neurobehavioral outcomes (e.g., ADHD/ASD-related symptoms, emotional and behavioral difficulties, and social functioning). Padovani et al. highlighted that PBTS treated with cranial radiation therapy (CRT) often go on to develop neurocognitive deficits, which could lead to impaired memory, attention, and processing speed, and poor academic achievement [13]. Other studies have found that the radiation dosage, the brain volume irradiated, and age at treatment / remission duration [11] were predictors of neurocognitive outcomes. Recent studies have suggested that genetic factors [14, 15] also contribute to increased susceptibility to neurocognitive declines in PBTS. However, it remains unclear whether similar risk factors would predispose PBTS to neurobehavioral impairments.

Early childhood is a critical period for the development of social, emotional, and behavioral functioning. Given the increased vulnerability of the developing brain and the disrupted learning opportunities experienced by PBTS, we hypothesize that PBTS are at risk of neurobehavioral impairment. In this study, we investigated the prevalence and symptom burden of ADHD, ASD, and emotional and behavioral difficulties among PBTS. Additionally, we explored both patient-related and treatment-related factors that may predispose PBTS to neurobehavioral impairments.

## Methods

This study consisted of two parts: (1) we conducted a retrospective cohort study reviewing the medical records of PBTS from our territory-wide pediatric cancer registry database, and (2) we conducted a longitudinal cohort study investigating the neurobehavioral outcomes of PBTS identified from the database. All research methods were conducted in accordance with the Declaration of Helsinki. The study was approved by the Institutional Review Board of The University of Hong Kong/ Hospital Authority Hong Kong West Cluster (UW20-455) and Hospital Authority Central Institutional Review Board (HKCH-REC-2021-045).

### Retrospective cohort study

The Hong Kong Paediatric Haematology Oncology Study Group (HKPHOSG) database is a territory-wide pediatric cancer registry established since 2000. We included survivors of brain tumors who had been diagnosed at least two years prior, had completed their treatment, and showed no evidence of recurrence at the time of data collection. Children and adolescents with a brain tumor diagnosis (*n* = 299) from Jan 2005 to October 2022 were identified from the HKPHOSG database. Exclusion criteria included patients who passed away, without a complete medical record due to emigration or treatment outside Hong Kong and loss to follow-up.

The electronic medical records of PBTS were reviewed for treatment-related information and diagnosis of ADHD, ASD, and intellectual developmental disabilities (IDD) two years post diagnosis without evidence of relapse. Records relating to visits to healthcare professionals (child psychiatrists, developmental behavioral pediatricians, or psychologists) were also reviewed for the management of psychiatric symptoms such as depression, anxiety, or stress. ADHD and ASD were diagnosed based on diagnostic interviews and clinical evaluation conducted by either a child psychiatrist, developmental behavioral pediatrician, or psychologist. Diagnoses were made according to theDiagnostic and Statistical Manual of Mental Disorders, Fourth Edition (DSM-IV) criteria from 2000 to 2013 or based on the latest DSM-V criteria since 2014. Confirmatory diagnostic evaluations using Autism Diagnostic Observation Schedule (ADOS) or Autism Diagnostic Interview – Revised (ADI-R) would be conducted for children/ adolescents with suspected autism. The Strengths and Weaknesses of ADHD Symptoms and Normal Behavior Scale (SWAN) rating scale would be used as part of the diagnostic workup for children with ADHD. A diagnosis of IDD was based on an intelligence quotient (IQ) < 70 measured by the Wechsler Intelligence Scale for children – fourth edition (WISC-IV HK) and assessment for deficits in adaptive functioning by a psychologist. Endocrine dysfunction was identified based on relevant ICD-coded diagnoses and supporting clinical documentation. We considered endocrine diagnoses within the endocrine disease chapters of ICD-9-CM and ICD-10-CM, focusing on thyroid, pituitary, adrenal, gonadal/pubertal, calcium/parathyroid, pancreatic endocrine, and hypothalamic–pituitary disorders, together with clinically documented endocrine sequelae such as growth hormone deficiency, diabetes insipidus, hypogonadism, precocious puberty, delayed puberty, adrenal insufficiency, hypothyroidism, hyperthyroidism, and panhypopituitarism. Follow-up duration was defined as the period from the date of diagnosis to the date of retrieval of the patients’ medical records. Age at follow-up was defined as the survivor’s age on the date of retrieval of the patient’s medical records. The prevalence of ASD and ADHD in the Hong Kong population was estimated based on the electronic medical records retrieved from the Clinical Data Analysis and Reporting System (CDARS). Detailed methods are documented in the Supplementary material.

### Survey study on the mental wellbeing of PBTS

A detailed follow-up survey on the wellbeing of PBTS and their parents was conducted among parents. After explaining the study’s purpose and data privacy policies to parents/patients by telephone or during clinic visits, an electronic questionnaire had been sent to them. Participants aged ≥ 16 years completed a self-report questionnaire. For all participants aged ≤ 24 years, a parent-report questionnaire was completed by the parent. For participants aged 16–24 years with two available sources of data (for the same measurement), the self-report served as the primary measure, with the parent-report included in the secondary/sensitivity analyses. Written informed consent was obtained from the participants who agreed to complete the questionnaire.

### Measurements

Depression Anxiety Stress Scale–21 (DASS-21) is a 21-item self-report measure with three 7-item subscales assessing depression, anxiety, and stress over the past week [16]. The Chinese version of DASS-21 was used in our study. Parental Stress Scale (PSS) is a questionnaire comprised of 18 questions assessing parents’ feelings about their parenting role in terms of both the positive and negative aspects of parenthood. It was developed by Judy Berry and Warren Jones in 1995 [17]. The overall score ranges from 18 to 90, with higher marks indicating higher levels of parental stress. A validated Chinese version was used in this study [18]. Strengths and Weaknesses of ADHD symptoms and Normal behavior (SWAN) Rating Scale was used to screen symptoms of ADHD [19]. It provides an in-depth understanding of the types of ADHD: inattention, hyperactivity, or combined type. A validated Chinese version of the SWAN questionnaire was used in our study [20]. Autism Spectrum Quotient (AQ) consists of three versions for screening symptoms of ASD: the child version for children aged 4–11 years, the adolescent version for children aged 12–15 years, and a self-report version for children aged 16 or above. The validated Chinese version of AQ was used in our study [21, 22]. Emotional and behavioral difficulties. Strengths and Difficulties Questionnaire (SDQ) is a screening tool for behavioral issues in children and adolescents [23]. Five aspects were evaluated: emotional symptoms, conduct problems, hyperactivity/inattention, peer relationship problems and prosocial behavior issues [23]. The Pediatric Quality of Life Inventory (PedsQL) is a modular approach assessing children’s health-related quality of life. In our study, the PedsQL-Cancer Module (Cantonese version) was used to evaluate the acute and chronic health conditions of PBTS, including pain, vomiting, anxiousness caused by medical procedure or treatment procedure, worry, cognition, self-perception. and communication [24]. Sleep was assessed using two indicators: (1) overall sleep quality, rated on a five-point Likert scale by parent- or self-report, and (2) age-adjusted sleep deprivation, defined as nightly sleep duration below the lower bound of NIH age-specific recommendations [25] and coded as 1 if below the recommended threshold and 0 otherwise. As a pragmatic dimension-reduction approach, these two standardized indicators were combined using principal component analysis, and the first principal component was retained as a composite sleep score, with higher values indicating poorer sleep, see Supplementary Table 4. These indicators were combined via principal component analysis (PCA) to yield a single sleep quality score. Family socioeconomic status (SES) was summarized as a composite z-score derived from a PCA of monthly household income, per-capita living space, parental education, and receipt of social benefits, see Supplementary Table 5.

### Statistical analysis

Descriptive statistics were calculated for all variables. Group differences in the categorical variables were tested with Pearson’s χ² test (Fisher’s exact test for any expected cell count < 5). The comparisons of scale scores with Hong Kong reference samples were conducted using independent-samples Welch’s t-tests, with Cohen’s d as the effect size.

For survey-based comparisons, PBTS scores were benchmarked against previously published Hong Kong-based reference/community cohorts. Children aged 2–12 years were compared with a large population-representative Hong Kong cohort (*n* = 29,202 families) [26]. Adolescents aged 13–16 years were compared with a Hong Kong community sample [27] individually matched 1:5 by age and sex (*n* = 146), and young adults aged 17–34 years with a Hong Kong community sample individually matched 1:5 by age, sex, and respondent type (*n* = 190). Means and standard deviations were obtained from these published cohorts and matched subsamples. Group differences were assessed using Welch’s t-tests and summarized using Hedges’ g. These comparisons should be interpreted as benchmark comparisons rather than fully adjusted between-group contrasts.

Relative risks (RRs) for clinical diagnoses were estimated using log binomial regression. Associations between risk/protective factors and continuous outcomes (SDQ, PedsQL, SWAN, AQ, and DASS) were examined by multivariable linear regression, adjusting for age at diagnosis, follow-up duration, sex, and socioeconomic status. To harmonize age-specific forms (AQ child < 12 years, adolescent 13–15 years, adult ≥ 16 years), we standardized each measure within its age group using z-scores (z = [raw score − age-group mean] / age-group SD). This standardization preserved directional consistency (higher z indicates greater symptoms burden) and allowed pooling across forms. The resulting pooled z-scores were used exclusively for the multivariate linear regression risk-factor analyses, whereas all other summaries (means, SDs) are presented in the original metrics. Regression results were reported as standardized β coefficients with standard errors (SE). All tests were two-sided with α = 0.05.

The analyses presented in Tables 1, 2 and 3 were treated as the principal hypothesis-driven analyses, as they addressed the primary study aims of describing neurobehavioral outcomes in PBTS and comparing symptom burden with Hong Kong reference/community samples. By contrast, the multivariable regression analyses examining multiple candidate predictors across questionnaire-based outcomes, as well as subgroup analyses such as the tumor-location analyses, were considered exploratory. Given the modest survey sample size relative to the number of outcomes and predictors examined, these exploratory analyses were interpreted cautiously, and false discovery rate (FDR) correction was applied.

**Table 1:** Demographic details of pediatric brain tumor survivors

Variable	Category	ADHD		ASD		Intellectual disability		Emotional difficulties (Post-treatment)	
Diagnosis		No (*n* = 245)	Yes (*n* = 29)	No (*n* = 255)	Yes (*n* = 19)	No (*n* = 262)	Yes (*n* = 12)	No (*n* = 241)	Yes (*n* = 33)
Mean age at diagnosis	—	9.88 ± 5.66	6.45 ± 4.32** ^**1**^	9.69 ± 5.62	7.28 ± 5.38	9.59 ± 5.64	8.03 ± 5.43	9.47 ± 5.70	9.92 ± 5.11
Mean duration of follow-up	—	8.21 ± 4.23	6.71 ± 3.21*	8.10 ± 4.21	7.40 ± 3.46	8.05 ± 4.06	8.18 ± 6.15	7.84 ± 3.99	9.64 ± 5.01
Sex	Male	147 (90.74%)	15 (9.26%)	151 (93.21%)	11 (6.79%)	157 (96.91%)	5 (3.09%)	142 (87.65%)	20 (12.35%)
	Female	98 (87.50%)	14 (12.50%)	104 (92.86%)	8 (7.14%)	105 (93.75%)	7 (6.25%)	99 (88.39%)	13 (11.61%)
Follow-up time	< 5 years	65 (85.53%)	11 (14.47%)	72 (94.74%)	4 (5.26%)	73 (96.05%)	3 (3.95%)	72 (94.74%)	4 (5.26%)
	5–<10 years	102 (88.70%)	13 (11.30%)	104 (90.43%)	11 (9.57%)	108 (93.91%)	7 (6.09%)	100 (86.96%)	15 (13.04%)
	10–<15 years	57 (91.94%)	5 (8.06%)	59 (95.16%)	3 (4.84%)	61 (98.39%)	1 (1.61%)	51 (82.26%)	11 (17.74%)
	≥ 15 years	21 (100.00%)	0 (0.00%)	20 (95.24%)	1 (4.76%)	20 (95.24%)	1 (4.76%)	18 (85.71%)	3 (14.29%)
Surgery	No	48 (87.27%)	7 (12.73%)	50 (90.91%)	5 (9.09%)	54 (98.18%)	1 (1.82%)	49 (89.09%)	6 (10.91%)
	Yes	197 (89.95%)	22 (10.05%)	205 (93.61%)	14 (6.39%)	208 (94.98%)	11 (5.02%)	192 (87.67%)	27 (12.33%)
Chemotherapy	No	86 (86.87%)	13 (13.13%)	90 (90.91%)	9 (9.09%)	93 (93.94%)	6 (6.06%)	93 (93.94%)	6 (6.06%)
	Yes	159 (90.86%)	16 (9.14%)	165 (94.29%)	10 (5.71%)	169 (96.57%)	6 (3.43%)	148 (84.57%)	27 (15.43%)*
Radiotherapy (RT)^2^	No	105 (86.07%)	17 (13.93%)	112 (91.80%)	10 (8.20%)	114 (93.44%)	8 (6.56%)	114 (93.44%)	8 (6.56%)
	Yes	137 (91.95%)	12 (8.05%)	140 (93.96%)	9 (6.04%)	145 (97.32%)	4 (2.68%)	125 (83.89%)	24 (16.11%)*
RT dose range^2^	≤ 30 Gy	35 (87.50%)	5 (12.50%)	37 (92.50%)	3 (7.50%)	40 (100.00%)	0 (0.00%)	34 (85.00%)	6 (15.00%)
	> 30–≤50 Gy	42 (95.45%)	2 (4.55%)	42 (95.45%)	2 (4.55%)	44 (100.00%)	0 (0.00%)	34 (77.27%)	10 (22.73%)
	> 50 Gy	50 (92.59%)	4 (7.41%)	50 (92.59%)	4 (7.41%)	51 (94.44%)	3 (5.56%)	48 (88.89%)	6 (11.11%)
Epileptic seizure	No	210 (90.91%)	21 (9.09%)	216 (93.51%)	15 (6.49%)	226 (97.84%)	5 (2.16%)	200 (86.58%)	31 (13.42%)
	Yes	35 (81.40%)	8 (18.60%)	39 (90.70%)	4 (9.30%)	36 (83.72%)	7 (16.28%)**	41 (95.35%)	2 (4.65%)
Hydrocephalus	No	116 (91.34%)	11 (8.66%)	120 (94.49%)	7 (5.51%)	123 (96.85%)	4 (3.15%)	113 (88.98%)	14 (11.02%)
	Yes	129 (87.76%)	18 (12.24%)	135 (91.84%)	12 (8.16%)	139 (94.56%)	8 (5.44%)	128 (87.07%)	19 (12.93%)
Tumor location^2^	Supratentorial	78 (86.67%)	12 (13.33%)	85 (94.44%)	5 (5.56%)	85 (94.44%)	5 (5.56%)	82 (91.11%)	8 (8.89%)
	Suprasellar	66 (91.67%)	6 (8.33%)	70 (97.22%)	2 (2.78%)	68 (94.44%)	4 (5.56%)	62 (86.11%)	10 (13.89%)
Tumor location	Infratentorial	87 (91.58%)	8 (8.42%)	85 (89.47%)	10 (10.53%)	93 (97.89%)	2 (2.11%)	81 (85.26%)	14 (14.74%)
	Multiple	10 (76.92%)	3 (23.08%)	12 (92.31%)	1 (7.69%)	12 (92.31%)	1 (7.69%)	12 (92.31%)	1 (7.69%)
Diagnosis	Craniopharyngioma	14 (87.50%)	2 (12.50%)	15 (93.75%)	1 (6.25%)	16 (100.00%)	0 (0.00%)	15 (93.75%)	1 (6.25%)
	DNET	7 (77.78%)	2 (22.22%)	8 (88.89%)	1 (11.11%)	7 (77.78%)	2 (22.22%)	9 (100.00%)	0 (0.00%)
	Ependymoma	15 (100.00%)	0 (0.00%)	13 (86.67%)	2 (13.33%)	15 (100.00%)	0 (0.00%)	15 (100.00%)	0 (0.00%)
	Ganglioglioma	7 (87.50%)	1 (12.50%)	8 (100.00%)	0 (0.00%)	7 (87.50%)	1 (12.50%)	8 (100.00%)	0 (0.00%)
	Germ cell tumor	74 (92.50%)	6 (7.50%)	75 (93.75%)	5 (6.25%)	78 (97.50%)	2 (2.50%)	68 (85.00%)	12 (15.00%)
	Low-grade astrocytoma	47 (85.45%)	8 (14.55%)	48 (87.27%)	7 (12.73%)	52 (94.55%)	3 (5.45%)	50 (90.91%)	5 (9.09%)
	Medulloblastoma	33 (91.67%)	3 (8.33%)	34 (94.44%)	2 (5.56%)	35 (97.22%)	1 (2.78%)	29 (80.56%)	7 (19.44%)
	Meningioma	6 (85.71%)	1 (14.29%)	7 (100.00%)	0 (0.00%)	6 (85.71%)	1 (14.29%)	4 (57.14%)	3 (42.86%)*
	Others	36 (87.80%)	5 (12.20%)	40 (97.56%)	1 (2.44%)	40 (97.56%)	1 (2.44%)	37 (90.24%)	4 (9.76%)
	High-grade astrocytoma	6 (85.71%)	1 (14.29%)	7 (100.00%)	0 (0.00%)	6 (85.71%)	1 (14.29%)	6 (85.71%)	1 (14.29%)
Deceased	No (alive)	237 (89.43%)	28 (10.57%)	247 (93.21%)	18 (6.79%)	253 (95.47%)	12 (4.53%)	234 (88.30%)	31 (11.70%)
	Yes (deceased)	8 (88.89%)	1 (11.11%)	8 (88.89%)	1 (11.11%)	9 (100.00%)	0 (0.00%)	7 (77.78%)	2 (22.22%)

Note. Values shown are mean +/- SD or n (%). (1) ** *p* < .01, * *p* < .01, p-values were calculated using Welch t-test for comparisons of group means and binary logistic regression for binary outcomes adjusted for follow-up time. (2) For some cases, RT status (*n* = 3), RT range (*n* = 11), and tumour location (*n* = 4) could not be reliably determined from the clinical record and were excluded from the table. *Abbreviations*: ADHD, attention-deficit/hyperactivity disorder; ASD, autism spectrum disorder; PBTS, pediatric brain tumor survivors; CI, confidence interval; RT, radiotherapy; Gy, gray; DNET, dysembryoplastic neuroepithelial tumor

**Table 2:** Risk factors of neurobehavioral impairment (ADHD & ASD) and emotional problems among PBTS post brain tumor treatment (*N* = 274)

Model 1 Adjusted for follow-up time and sex						
	ADHD or ASD diagnosis post brain tumor treatment			Emotional problems post brain tumor treatment		
Risk factors	RR	95% CI	p	RR	95% CI	p
Age at diagnosis (years)	**0.90**	**(0.85**,**0.96)**	**< 0.001**	1.01	(0.95, 1.07)	0.797
Chemotherapy	0.63	(0.34,1.18)	0.141	**2.77**	**(1.22**,** 7.43)**	**0.013**
Radiotherapy	0.72	(0.39,1.32)	0.295	**2.14**	**(1.03**,** 4.86)**	**0.042**

Model 2 Adjusted for follow-up time, sex, tumor type, surgery status, seizure history endocrine dysfunction and hydrocephalus						
	ADHD or ASD diagnosis			Emotional problems post brain tumor treatment		
Risk factors	RR	95% CI	p	RR	95% CI	p
Age at diagnosis (years)	**0.89**	**(0.85**,** 0.92)**	**< 0.001**	0.99	(0.93, 1.05)	0.731
Chemotherapy	0.77	(0.41, 1.43)	0.405	1.65	(0.63, 4.29)	0.305
Radiotherapy	0.78	(0.36, 1.69)	0.532	2.25	(0.79, 6.40)	0.127

**Table 3:** Comparison of SDQ and PSS between PBTS children and TD control samples

(a) Comparison of outcomes for children (parent-report, age 3–12)					
Measure	Mean (SD) of PBTS (age 3–12, *n* = 42)		Mean (SD) Representative Hong Kong Sample^3^ (age 3–12, *n* = 29,202)	Hedge‘s *g*^1^(95% CI)	p value^2^
SDQ: Emotional symptoms	4.62 (2.85)		2.09 (1.77)	**1.43 (1.12**,** 1.73)**	**< 0.001**
SDQ: Conduct problems	2.74 (2.01)		2.27 (1.58)	0.30 (-0.01, 0.60)	0.138
SDQ: Hyperactivity	3.86 (2.28)		4.84 (2.22)	**-0.44 (-0.74**,** -0.14)**	**0.008**
SDQ: Peer relationship problems	3.17 (2.07)		2.89 (1.74)	0.16 (-0.14, 0.46)	0.386
SDQ: Total difficulties	14.38 (6.92)		12.09 (5.42)	**0.42 (0.12**,** 0.72)**	**0.038**
PSS: Parental stress	56.09 (11.87)	49.38 (10.48)		**0.64 (0.34**,** 0.94)**	**0.001**

(b) Comparison of outcomes for adolescents (parent-report, age 13–16)					
Measure	Mean (SD) of PBTS (age 13–16, *n* = 27)	Matched Hong Kong Community Sample^4^ (age 13–16, *n* = 146)		Hedge‘s *g*^1^(95% CI)	p value
SDQ: Emotional symptoms	5.15 (3.72)	2.04 (1.77)		**1.42 (0.98**,** 1.86)**	**< 0.001**
SDQ: Conduct problems	3.26 (2.47)	2.10 (1.76)		**0.61 (0.20**,** 1.03)**	**0.026**
SDQ: Hyperactivity	4.22 (2.72)	3.53 (2.66)		0.26 (-0.15, 0.67)	0.232
SDQ: Peer relationship problems	4.00 (2.09)	2.89 (1.42)		**0.72 (0.30**,** 1.13)**	**0.013**
SDQ: Total difficulties	16.63 (9.00)	10.56 (5.77)		**0.95 (0.53**,** 1.37)**	**0.002**

(c) Comparison of outcomes for young adults (parent-report & self-report, age 17–34)					
Measure	Mean (SD) of PBTS (age 17–34, *n* = 38)	Matched Hong Kong Community Sample^5^ (age 17–34, *n* = 190)		Hedge‘s *g*^1^(95% CI)	p value
SDQ: Emotional symptoms	4.89 (3.21)	3.67 (2.61)		**0.45 (0.10**,** 0.80)**	**0.033**
SDQ: Conduct problems	2.61 (1.82)	2.31 (1.35)		0.21 (-0.14, 0.56)	0.340
SDQ: Hyperactivity	3.39 (2.49)	4.47 (1.99)		**-0.52 (-0.87**,** -0.17)**	**0.015**
SDQ: Peer relationship problems	4.55 (2.20)	3.61 (1.67)		**0.53 (0.18**,** 0.88)**	**0.016**
SDQ: Total difficulties	15.45 (7.42)	14.06 (5.86)		0.23 (-0.12, 0.57)	0.282

1 Hedges’ g (bias-corrected Cohen’s d) was used to obtain an approximate unbiased effect size given the modest, unequal group sizes

2 p values estimated based on Welch t-test

3 This cohort was originally reported in (Tso et al., 2022) [26]

4 This cohort was adapted from the sample originally reported in (Tso et al., 2024) [27], 1:5 age and gender matched

5 This cohort was adapted from the sample originally reported in (Tso et al., 2024) [27], 1:5 age, gender, and rater matched

SDQ: Strengths and Difficulties Questionnaire; PSS: Parental Stress scale; PBTS: Pediatric Brain Tumor Survivors; TD: Typical Development

Missing data were handled using complete-case analysis for each specific model. Analyses were conducted in R version 4.0.9 and Python 3.12 using custom scripts.

## Results

### Regional representative cohort

We reviewed the clinical records of 299 PBTS diagnosed between Jan 2005 and Oct 2020 within the HKPHOSG database. Twenty-five were excluded due to incomplete or unretrievable medical records, leaving 274 records for chart review.

### Clinical outcomes

#### Participant characteristics

The mean age at brain tumor diagnosis was 9.52 ± 5.63 years, mean age at follow-up was 17.57 ± 7.21 years, and mean duration of follow-up was 8.05 ± 4.16 years. The most common types of brain tumor were germ cell tumor (GCT) and astrocytoma (Table 1). Overall, 33 cases (12.0%) had symptoms of anxiety, depression, or stress requiring psychological support from healthcare professionals (psychiatrists/ psychologists/ pediatricians).

#### Risk factors for PBTS with ASD/ ADHD

The PBTS cohort showed significantly higher prevalence of both ADHD and ASD compared to the Hong Kong general population birth cohort (2004–2018). The prevalence of ADHD in PBTS was 10.58% (29/274) compared to 4.86% (25,930/532,881) in the general population, corresponding to an adjusted increased risk (aRR = 1.66, 95% CI 1.14–2.43; *p* = .009). The prevalence of ASD in PBTS was 6.93% (19/274) (aRR = 3.95, 95% CI 2.47–6.23; *p* < .001) compared to 2.27% (12,120/532,881) in the general population, adjusted for age at diagnosis, follow-up duration, sex and socioeconomic status (Supplementary Table 1). 6 (2.2%) PBTS had both ADHD and ASD diagnoses. Among the PBTS group, 22/274 (8.03%) were diagnosed of ADHD after the brain tumor diagnosis, and 10/274 (3.65%) were diagnosed with ASD after the brain tumor diagnosis.

Further analyses were conducted to identify risk factors for neurobehavioral outcomes (Table 2). In Model 1, adjusted for follow-up time and sex, older age at diagnosis was significantly associated with a lower risk of post-treatment ADHD/ASD, corresponding to an approximately 10% reduction in risk per additional year of age at diagnosis (RR = 0.90, 95% CI 0.85–0.96, *p* < .001), whereas chemotherapy and radiotherapy were not significantly associated with ADHD/ASD. In Model 2, additionally adjusted for tumor type, surgery status, seizure history, endocrine dysfunction, and hydrocephalus, the association between older age at diagnosis and lower risk of ADHD/ASD remained robust (RR = 0.89, 95% CI 0.85–0.92, *p* < .001). This association remained robust in a supplementary sensitivity analysis that included diagnoses made both before and after brain tumor diagnosis (Supplementary Table 6).

For emotional problems requiring psychological support, chemotherapy and radiotherapy were associated with increased risk in Model 1 (chemotherapy: RR = 2.77, 95% CI 1.22–7.43, *p* = .013; radiotherapy: RR = 2.14, 95% CI 1.03–4.86, *p* = .042). However, these associations were attenuated and no longer statistically significant in Model 2 after additional adjustment for tumor type, surgery status, seizure history, endocrine dysfunction, and hydrocephalus. These findings should therefore be interpreted cautiously.

### Mental wellbeing of PBTS from the proxy or self-report surveys

**Fig. 1:**
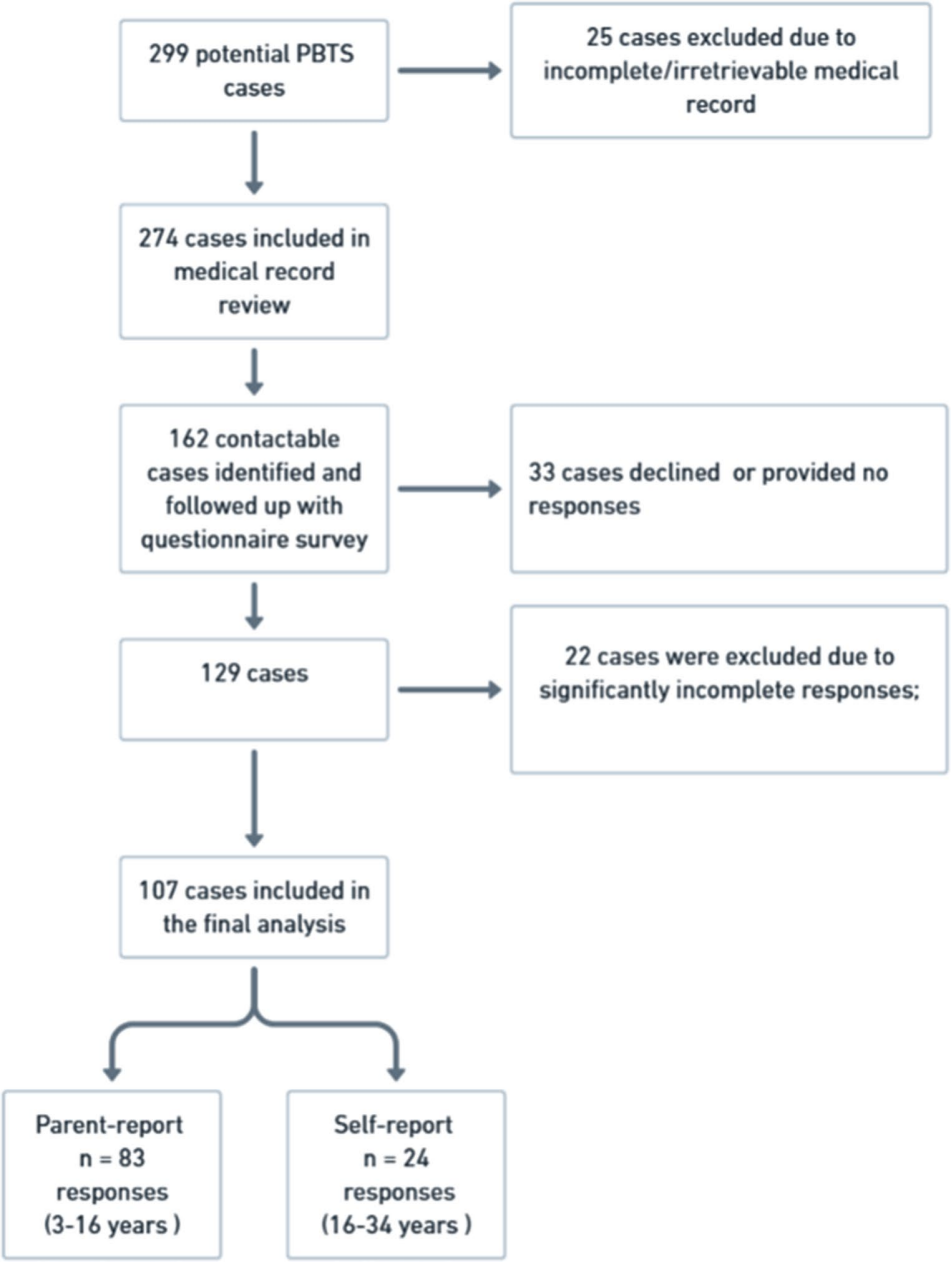
Study flow

Among the 274 PBTS identified from the HKPHOSG database, 162 were contactable and invited to complete the questionnaires, of whom 33 declined or did not respond, giving a total of 129 completed questionnaires. After excluding 22 cases with incomplete questionnaires which not all items were completed, 107 cases remained for the analysis. Among the 16–24 age group, 19 had both self- and parent-reported responses. The self-report surveys served as the primary measure, while the parent-report surveys were included in secondary/sensitivity analyses. The final analytic samples comprised 83 parent-report responses and 24 self-report responses (Fig. 1).

To assess potential response bias, we compared key characteristics between survey responders and non-responders within the registry cohort (Supplementary Table 2). Responders and non-responders did not differ significantly in age at diagnosis, diagnosis category, or treatment exposures including surgery, chemotherapy, and radiotherapy (all *p* > .05). However, responders were younger at follow-up/current age and had a shorter time since diagnosis than non-responders (both *p* < .001).

In the current cohort, PBTS showed consistently higher SDQ emotional symptoms and peer relationship problems across childhood, adolescence, and young adulthood than the other Hong Kong representative comparison groups [26, 28], with additional differences observed in conduct problems, hyperactivity, total difficulties, and parental stress (Table 3). Compared with the matched Hong Kong reference/community samples, PBTS showed higher emotional symptoms across children (standardized mean difference [SMD] = 1.43, *p* < .001), adolescents (SMD = 1.42, *p* < .001), and young adults (SMD = 0.45, *p* = .033). hey also had higher total difficulties in children (SMD = 0.42, *p* = .038) and adolescents (SMD = 0.95, *p* = .002), more peer problems in adolescents (SMD = 0.72, *p* = .013) and young adults (SMD = 0.53, *p* = .016), and higher parental stress in caregivers of children (SMD = 0.64, *p* = .001). Adolescents additionally showed more conduct problems (SMD = 0.61, *p* = .026), whereas children (SMD = − 0.44, *p* = .008) and young adults (SMD = − 0.52, *p* = .015) showed lower hyperactivity scores than their respective age-matched Hong Kong reference/community groups.

The mean quality of life rating for PBTS based on the PedsQL cancer module was less than 80 for all domains, with the lowest subdomain being cognitive problems. The averaged PedsQL total rating was highly associated with emotional and behavioral problems, as measured by SDQ (β = −0.44 [0.10], *p* < .001, adjusted for age of diagnosis, follow-up time, sex and SES). The results are summarized in Supplementary Table 3.

### Risk and protective factors for mental wellbeing in PBTS

In exploratory regression analyses, older age at diagnosis was associated with fewer ADHD symptoms (SWAN; β = −0.27 [0.10], *p* = .005, q = 0.008), and better sleep quality was associated with fewer ADHD symptoms (SWAN; β = −0.30 [0.09], *p* = .002, q = 0.006) and lower affective symptoms (DASS; β = −0.57 [0.18], *p* = .005, q = 0.015). Higher RT dose was associated with poorer quality of life at the unadjusted level (PedsQL; β = −0.20 [0.10], *p* = .041), but this association did not remain significant after FDR correction (q = 0.123) (Table 4).

**Table 4:** Association between risk factors and neurodevelopmental, emotional, and behavioral problems in PBTS (*n* = 107)

	Age at Diagnosis	RT dosage	Sleep quality
SDQ Total	β=-0.07 (SE = 0.09); *p* = .492, FDR q = 0.738	β = 0.03 (SE = 0.10); *p* = .744, FDR q = 0.744	β=-0.16 (SE = 0.10); *p*=.110, FDR q = 0.330
PedsQL Total	β = 0.11 (SE = 0.10); *p* = .261, FDR q = 0.261	β=-0.20 (SE = 0.10); *p* = .041*, FDR q = 0.123	β = 0.16 (SE = 0.10); *p*=.121, FDR q = 0.181
SWAN Total	**β=-0.27 (SE = 0.10);** *p* = .005** FDR q = 0.008**	β=-0.00 (SE = 0.10); *p* = .987 FDR q = 0.987	**β=-0.30 (SE = 0.09);** *p*=.002** FDR q = 0.006**
AQ Total	β=-0.22 (SE = 0.11); *p* = .054, FDR q = 0.162	β=-0.04 (SE = 0.11); *p* = .709 FDR q = 709	β=-0.11 (SE = 0.11); *p*=.321 FDR q = 0.482
DASS Total	β=-0.35 (SE = 0.21); *p* = .107, FDR q= 0.161	β=-0.21 (SE = 0.20); *p* = .310 FDR q = 0.310	**β=-0.57 (SE = 0.18);** *p*=.005** FDR q = 0.015*

Note. Results were adjusted by age of diagnosis, follow-up time, sex, and SES, standardised regression coefficient beta was reported. *p/q < 0.05; ** p/q < 0.01

A further exploratory analysis was conducted to investigate the potential protective and risk factors for the behavioral and emotional difficulties in PBTS (Supplementary Fig. 1). Better sleep was associated with lower anxiety, depression, and stress scores on the DASS symptom scales. After FDR correction, the associations remained significant between sleep and anxiety (β = −0.51 [0.19], *p* = .013, q = 0.047), depression (β = −0.42 [0.14], *p* = .009, q = 0.047), stress (β = −0.49 [0.18], *p* = .012, q = 0.047). Older age at diagnosis also showed protective associations on the DASS depression scale that remained after FDR (β = −0.48 [0.15], *p* = .004, q = 0.047).

### Tumor location and emotional/behavioral symptoms

An exploratory analysis was conducted to examine the impact of tumor location on neurobehavioral symptoms burden in PBTS (Supplementary Fig. 2). PBTS with supratentorial tumors were more likely to have higher scores in the SDQ hyperactivity domain than those with tumors in other regions (*p* = .005, adjusted for age at diagnosis and follow-up time).

## Discussion

This is one of the first comprehensive studies of a regionally representative cohort evaluating the risk and protective factors affecting neurobehavioral outcomes and mental well-being in survivors of pediatric brain tumors. Specifically, the present study offers novel insights into the complex relationship between ASD and pediatric brain tumor diagnoses. We found that PBTS were associated with ASD diagnosis. However, our study identified a proportion of PBTS cases that were labeled with ASD after tumor treatment, which contrasts with a recent study showing an elevated risk of ASD diagnoses prior to diagnosis among patients with intracranial germinoma [29]. Also, we observed consistently elevated risk of ADHD, with 10.6% PBTS diagnosed with ADHD, which is consistent with the existing literature [10, 30].

A recent study by Liu et al. reported 6% of germinoma patients with an ASD diagnosis prior to their cancer diagnosis had more than threefold increased risk of ASD than the general population [29]. Existing studies and case reports have suggested that genetic predisposition might be a major factor underlying the increased ASD risk observed in germinoma patients [31]. Also, most studies investigating the relationship between ASD and childhood brain tumors have focused on germinoma patients [29, 32], whereas our cohort revealed that ependymoma patients exhibited the highest rates of ASD diagnosis. Further research is needed to elucidate the mechanisms underlying the associations between ASD and specific subtypes of childhood brain tumors.

Our findings from the survey study revealed that adolescent and young adult brain tumor survivors have higher risk of emotional and peer-relationship problems. We postulate that in some PBTS with no genetic susceptibility to ASD, their social communication difficulties or autistic-features may stem from disruptions in social-affective brain networks [34] caused by the tumor or its treatment. One study showed that children with medulloblastoma and suffering from cerebellar mutism syndrome post-operatively might develop ASD-like network disturbance with communication and social deficits without repetitive behaviours [33]. These children might not fulfil the diagnostic criteria of ASD, yet they would benefit from social skills training and extra support for their autistic-like behaviors.

The increased risk of ADHD among PBTS was previously reported by Shabason et al. (2019), who found that 13.1% of PBTS had a documented ADHD diagnosis, with an additional 19.9% exhibiting ADHD symptoms without a formal diagnosis [34]. Meta-analyses have also documented elevated attentional problems in PBTS via both parent-reported behavioral ratings [35] and performance-based cognitive assessments [36]. Neurobiologically, PBTS frequently sustain focal and diffuse injury to the frontostriatal, frontoparietal and cerebello-thalamo-cortical circuits due to the primary tumor, surgery, and cranial RT/chemotherapy, resulting in white-matter loss, demyelination, and altered network topology and leading to ADHD-like deficits in attention and executive control [37–39]. Previous studies have also reported that ADHD/attention problems following pediatric brain tumor may be shaped by external factors such as age at diagnosis, tumor location, and treatment/brain injury burden [34, 40]. Consistent with these reports, we found that supratentorial tumors were associated with the risk of attention problems. Such supratentorial lesions and their treatment frequently affect fronto-striatal and fronto-parietal attention networks that have also been implicated in idiopathic ADHD and linked to attentional control problems in PBTS [30, 41].

Interestingly, in our PBTS cohort, we found that sleep is a novel and potentially modifiable factor of behavioral deficits, with better sleep predicting lower attentional and affective symptoms burden. Sleep quality is crucial for maintaining good mental health, and this seems particularly important for PBTS, whom a substantial proportion have clinically significant sleep problems or excessive daytime sleepiness associated with poorer executive functioning, fatigue, and reduced quality of life [42, 43]. Mechanistically, sleep disorders in this group are thought to arise from tumor- and treatment-related damage to hypothalamic and brainstem regions, endocrine and metabolic disturbances [44], and sleep-related breathing disorders, whereas the broader pediatric literature shows that short or poor-quality sleep adversely affects cognitive performance and emotional–behavioral regulation, thereby amplifying attentional and affective difficulties in vulnerable survivors [45].

Recent reviews agree that younger age at brain-tumor diagnosis/treatment is one of the most consistent risk factors for later neurocognitive problems in survivors, including lower IQ, processing speed, and working memory, especially in those treated before about 6–7 years and exposed to cranial radiotherapy or intensive multimodal therapy [36, 46, 47]. Yet, evidence linking younger age at diagnosis to later emotional/behavioral issues or peer relationship problems is scarce and mixed. Some longitudinal and survivorship studies showed that younger treatment age and longer time since treatment predict worse psychosocial adjustment and social functioning [48], whereas others reported no association between age at diagnosis and poor behavioural outcomes [49, 50]. Our study confirmed that younger age at diagnosis was associated with a higher risk of neurodevelopmental disorders. This is consistent with the vulnerability hypothesis that proposes tumor- and treatment-related insults in early childhood occurring during periods of rapid brain maturation disrupt ongoing processes such as synaptogenesis, myelination, and network specialization in fronto-limbic and cerebellar–cortical circuits, creating more diffuse and enduring alterations in brain architecture and connectivity, predisposing children to long-term cognitive, emotional, and social sequelae [51–53].

In our cohort, chemotherapy and radiotherapy were associated with a higher risk of emotional problems in the parsimonious model, but these associations were attenuated and no longer statistically significant after further adjustment for clinical comorbidities. This suggests that treatment-related associations with emotional problems should be interpreted cautiously, as they may partly reflect confounding by disease severity or other neurological/endocrine sequelae. Nevertheless, the direction of association is broadly consistent with prior reports [54, 55]. Both RT and chemotherapy are thought to induce white-matter injury, microvascular damage, neuroinflammation, and altered neurogenesis in fronto-limbic circuits, leading to disrupted connectivity, slower processing speed, and heightened stress reactivity that manifest as anxiety, depression, and emotional dysregulation [56, 57]. Peer and environmental factors further shape the social–affective outcomes, with poorer social-information processing and lower-quality peer relationships predicting worse trajectories in social competence, bullying, or social exclusion, exacerbating social withdrawal and distress [58, 59].

### Limitations

One potential limitation of this study is the sample size, which might constrain the study power. Although we used a regional population cohort, there are only around 30 new pediatric brain tumor cases diagnosed each year in Hong Kong, hence, the overall PBTS cohort is relatively small, especially for the surveyed cohort. Second, PBTS would have more frequent contact with healthcare providers. Therefore, surveillance bias is possible which would increase the chance of identifying PBTS with neurobehavioral impairment. Nevertheless, in Hong Kong, children and adolescents would have routine developmental or mental health surveillance by the Maternal & Child Health Centre (MCHC) or the Student Health Service (SHS) [60]. Educators also work closely with the healthcare system to facilitate diagnoses of ASD and ADHD which would reduce the chance of missed diagnoses in the general pediatric population. Third, the follow-up survey sample was also modest, although its findings were consistent with the clinical chart review. In addition, although survey responders and non-responders were broadly similar in diagnosis category and major treatment exposures, responders were younger at follow-up and had a shorter time since diagnosis, suggesting response bias and possible underrepresentation of older survivors and those further from diagnosis. This may limit generalizability. Moreover, the survey sample size was modest for the number of exploratory regression analyses performed. Although FDR correction was applied, these models may still have been underpowered and susceptible to overfitting, and some unadjusted associations did not remain significant after multiple-testing correction. Fourth, the survey component was cross-sectional and based on a single follow-up assessment, which precludes inference regarding temporal relationships or causality for the identified risk and protective factors Future studies should consider a longitudinal design to confirm and extend these findings. Fifth, sleep was not assessed using a validated sleep scale. Instead, we relied on two pragmatic indicators, overall sleep quality and age-adjusted sleep deprivation, which may not fully capture the multidimensional nature of sleep. Lastly, the survey findings may have been influenced by surveillance bias, as PBTS generally have more frequent medical follow-up than the general pediatric population, and by reliance on parent-/self-report measures, which may be subject to reporting bias. Furthermore, the absence of longitudinal behavioral assessments limited our ability to evaluate changes in neurobehavioral outcomes over time.

## Conclusions

Survivors of childhood brain tumors have increased risk of ADHD and poorer mental wellbeing. Significant association was found between PBTS and ASD diagnosis. We found that sleep is a potential protective factor against ADHD and emotional difficulties. These findings underscore the need for comprehensive survivorship care, which should include systematic, long-term surveillance of developmental, neurobehavioral, emotional, and sleep problems in PBTS. Professionals involved in childhood cancer care should emphasize the role of sleep and lifestyle measures, such as physical activity, in promoting well-being. Future longitudinal, multi-center studies are warranted to clarify the causal mechanisms, identify high-risk subgroups, and test targeted interventions—including sleep-focused and psychosocial programs—to improve long-term outcomes for this vulnerable population.

## Supplementary Information

Below is the link to the electronic supplementary material.

Supplementary Material 1

## Data Availability

Data will be made available upon reasonable request.
