# Supplementary material for: Neurobehavioral outcomes and associated risk factors in pediatric brain tumor survivors
Source: J Neurooncol. 2026 Apr 11;177(2):98. doi: 10.1007/s11060-026-05558-8 (PMC13070074; doi:10.1007/s11060-026-05558-8)
Supplement: Supplementary file 1 — Supplementary Material 1 [file 11060_2026_5558_MOESM1_ESM.docx]

**Supplementary Method**

**Concepts and definitions**

1. Persons with ASD refer to those who have been diagnosed with ASD by clinical assessment tests.
2. Persons with ADHD refer to those who have been diagnosed with ADHD by clinical assessment tests.
3. Persons with intellectual disability (ID) refer to those who have been diagnosed with ID (IQ ≤ 70) by clinical assessment tests.
4. Persons with emotional problems refer to those who self-reported or parent-reported to have anxiety, depression, or stress, and confirmed by a clinical psychologist or psychiatrist.

**Supplementary Table**

| Outcome | PBTS (n/N, %) | HK General Population (%) | aRR (95% CI) | p-value |
| --- | --- | --- | --- | --- |
| ADHD (total) | 29 / 274 (10.58%) | 25,930 / 532,881 (4.86%) | 1.660 (1.135–2.429) | .009 |
| ASD(total) | 19 / 274 (6.93%) | 12,120 / 532,881 (2.27%) | 3.946 (2.473–6.299) | < .001 |
| ADHD (Post-PBT treatment) | 22 / 274 (8.03%) |  |  |  |
| ASD (Post-PBT treatment) | 10 / 274 (3.65%) |  |  |  |

**Supplementary Table 1. Comparison of ADHD and ASD Prevalence in Pediatric Brain Tumor Survivors (PBTS) Versus the Hong Kong General Population (Births 2004–2018)**

Note. Relative Risk was adjusted PBT Pediatric Brain Tumor. General population prevalences were calculated from 532,881 births in Hong Kong (2004–2018)^[[1]](#footnote-1)^, excluding preterm deliveries and major perinatal/postnatal neurological complications. These children were followed up until Nov 2024. ADHD prevalence in the general population was 25,930/532,881 (4.86%); ASD prevalence was 12,120/532,881 (2.27%). Adjusted relative risk (aRR) and 95% CI were estimated via log binomial regression, adjusting for birth year and sex.

**Supplementary Table 2 Comparison of Clinical Characteristics Between Survey Responders and Non-Responders in the PBTS Cohort**

| **Characteristic** | **Level** | **Non-responders (n=167)** | **Responders (n=107)** | **P value** |
| --- | --- | --- | --- | --- |
| Age at diagnosis, years |  | 9.96 (5.51) | 8.93 (5.88) | 0.148 |
| Age at follow-up / current age, years | | 19.08 (7.11) | 15.78 (6.98) | <0.001 |
| Time since diagnosis, years | | 8.41 (4.16) | 6.10 (3.76) | <0.001 |
| Diagnosis category |  |  |  | 0.688 |
|  | Craniopharyngioma | 9 (5.4%) | 7 (6.5%) |  |
|  | DNET | 4 (2.4%) | 5 (4.7%) |  |
|  | Ependymoma | 8 (4.8%) | 7 (6.5%) |  |
|  | GCT | 54 (32.3%) | 26 (24.3%) |  |
|  | Ganglioglioma | 3 (1.8%) | 5 (4.7%) |  |
|  | High-grade astrocytoma | 5 (3.0%) | 2 (1.9%) |  |
|  | Low-grade astrocytoma | 32 (19.2%) | 23 (21.5%) |  |
|  | Medulloblastoma | 20 (12.0%) | 16 (15.0%) |  |
|  | Meningioma | 5 (3.0%) | 2 (1.9%) |  |
|  | Others | 27 (16.2%) | 14 (13.1%) |  |
| Surgery | Yes | 135 (80.8%) | 84 (78.5%) | 0.752 |
| Chemotherapy | Yes | 107 (64.1%) | 68 (63.6%) | 1.000 |
| Radiotherapy | Yes | 98 (58.7%) | 51 (47.7%) | 0.118 |

**Note:** Continuous variables are presented as mean (SD) and were compared between responders and non-responders using Welch’s t-test. Categorical variables are presented as n (%) and were compared using chi-square tests. Diagnosis was analysed as an overall categorical distribution across diagnosis groups.

**Supplementary Table 3. PedsQL Cancer module results in this cohort.**

| PedsQL Subdomains | Valid N | Mean (SD) |
| --- | --- | --- |
| Pain & Hurt | 104 | 76.92 (22.15) |
| Nausea | 104 | 77.93 (22.14) |
| Procedural Anxiety | 104 | 65.63 (29.46) |
| Treatment Anxiety | 104 | 74.36 (23.87) |
| Worry | 104 | 66.19 (24.60) |
| Cognitive Problems | 104 | 56.88 (20.74) |
| Appearance | 104 | 65.79 (24.85) |
| Communication | 104 | 66.43 (24.09) |
| Total Score | 104 | 68.26 (16.02) |

**Supplementary Table 4 Principal component analysis of the sleep composite**

| Variable | PC1 loading |
| --- | --- |
| Age-adjusted sleep deprivation | 0.707 |
| Overall sleep quality (5-point Likert scale) | -0.707 |
|  |  |
| PCA index | Value |
| Number of variables | 2 |
| Eigenvalue, PC1 | 1.03 |
| Variance explained by PC1 | 51.50% |

**Supplementary Table 5 Principal component analysis of the SES**

| Variable | PC1 loading |
| --- | --- |
| Monthly household income | 0.506 |
| Maternal education | 0.442 |
| Per-capita living space | 0.413 |
| Housing type | 0.406 |
| Paternal education | 0.403 |
| Receipt of social benefits | 0.226 |
| PCA index | Value |
| Number of variables | 6 |
| Eigenvalue, PC1 | 2.56 |
| Variance explained by PC1 | 42.28% |

**Supplementary Table 6. Risk factors of neurobehavioral impairment (ADHD & ASD) and emotional problems among PBTS (N =274)**

| **Model 1 Adjusted for follow-up time and sex** | | | | | | |
| --- | --- | --- | --- | --- | --- | --- |
|  | **ADHD or ASD diagnosis** | | | **Emotional problems** | | |
| Risk factors | RR | 95% CI | p | RR | 95% CI | p |
| Age at diagnosis (years) | 0.89 | (0.83, 0.96) | 0.001 | 1 | (0.95, 1.06) | 0.905 |
| Chemotherapy | 0.60 | (0.31, 1.19) | 0.146 | 2.88 | (1.24, 6.68) | 0.014 |
| Radiotherapy | 0.69 | (0.35, 1.35) | 0.278 | 2.35 | (1.10, 5.05) | 0.028 |
| **Model 2 Adjusted for follow-up time, sex, tumor type, surgery status,** **seizure history endocrine dysfunction and hydrocephalus** | | | | | | |
|  | **ADHD or ASD diagnosis** | | | **Emotional problems** | | |
| Risk factors | RR | 95% CI | p | RR | 95% CI | p |
| Age at diagnosis (years) | 0.86 | (0.82, 0.91) | <.001 | 1.01 | (0.96, 1.06) | 0.733 |
| Chemotherapy | 0.73 | (0.34, 1.53) | 0.4 | 1.91 | (0.77, 4.76) | 0.166 |
| Radiotherapy | 0.89 | (0.36, 2.23) | 0.811 | 2.39 | (0.85, 6.72) | 0.10 |

Note. This sensitivity analysis includes any history of ADHD/ASD and Emotional problems pre or post brain tumor diagnosis.


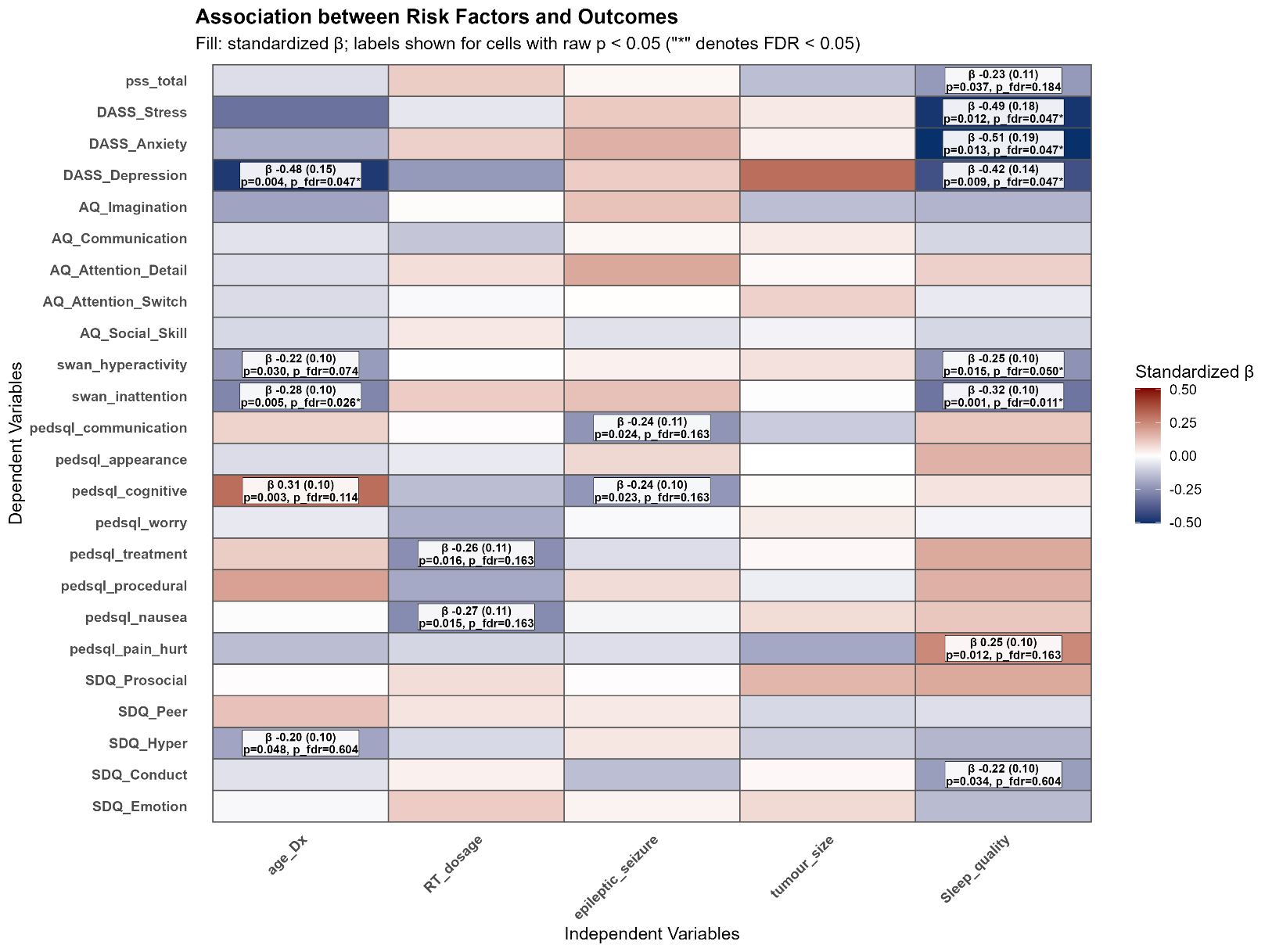


Supplementary Figure 1. Correlations between risk factors and subdomain scores of the outcome measures. Factors were mutually adjusted with additional adjustment for follow-up time.


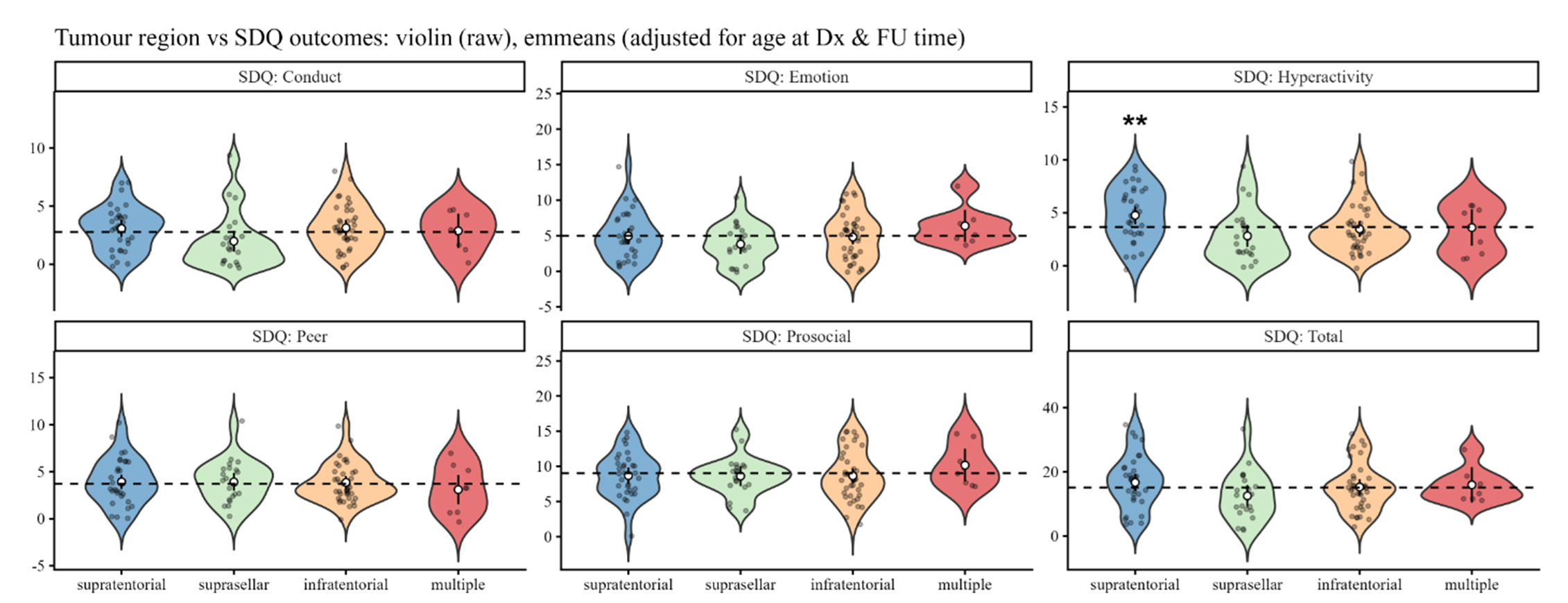
 Supplementary Figure 2. Comparison of behavioral and emotional difficulties in PBTS by tumor region. ** p <.01. p value estimated based on generalized linear model adjusted for diagnosis age and follow-up time. We fitted the four regions-vs.-the rest linear models with SDQ subdomains as the dependent variable and an indicator for each tumor region as the focal predictor, adjusting for age at diagnosis and follow-up time. Tumor region was treated categorically: for each run, the indicator was labeled 1 for the focal region and 0 otherwise. Supratentorial brain tumor were significantly associated with higher SDQ hyperactivity scores β = 1.483, SE = 0.518, p = .005.

1. This population-based birth cohort search analyzed electronic medical records retrieved from the Clinical Data Analysis and Reporting System (CDARS) in Hong Kong. The CDARS medical database was developed by the Hospital Authority, which is the only publicly funded health care provider in Hong Kong. The current study retrieved data on all term infants delivered in public hospitals between 1^st^ January 2004 and 31^st^ December 2018. Children with ASD with an ICD-9 diagnostic coding of “299.00” and children with ADHD with ICD-9 diagnostic coding of “314.00 attention-deficit and disruptive behavior disorders, predominantly inattentive type” and “314.01 attention-deficit and disruptive behavior disorders, combined type; or predominantly hyperactive-impulsive type” or prescribed medications for ADHD (i.e., methylphenidate or atomoxetine) were included in the study. In Hong Kong, children with ASD and ADHD have been clinically diagnosed by pediatricians, clinical psychologists, or child psychiatrists based on the Diagnostic and Statistical Manual of Mental Disorders, Fourth Edition (DSM-IV) from 2006 to 2013 and based on the latest DSM-V criteria since 2014. [↑](#footnote-ref-1)
